# Deletion of Budding Yeast *MAD2* Suppresses Clone-to-Clone Differences in Artificial Linear Chromosome Copy Numbers and Gives Rise to Higher Retention Rates

**DOI:** 10.3390/microorganisms8101495

**Published:** 2020-09-29

**Authors:** Scott C. Schuyler, Lin-Ing Wang, Yi-Shan Ding, Yi-Chieh Lee, Hsin-Yu Chen

**Affiliations:** 1 Department of Biomedical Sciences, College of Medicine, Chang Gung University, Kwei-Shan, Tao-Yuan 333, Taiwan; stickyorange@gmail.com (L.-I.W.); imysding@gmail.com (Y.-S.D.); yichieh4u26@gmail.com (Y.-C.L.); hsinyu.chen@mail.cgu.edu.tw (H.-Y.C.); 2 Division of Head and Neck Surgery, Department of Otolaryngology, Chang Gung Memorial Hospital, Kwei-Shan, Tao-Yuan 333, Taiwan

**Keywords:** budding yeast, *Saccharomyces cerevisiae*, chromosome, mitotic spindle checkpoint, Mitotic Arrest-Deficient 2 (*MAD2*), quantitative polymerase chain reaction (qPCR)

## Abstract

Our goal was to investigate the changes in artificial short-linear chromosome average copy numbers per cell arising from partial or full loss of Mitotic Arrest-Deficient 2 (*MAD2*) spindle checkpoint function in budding yeast *Saccharomyces cerevisiae*. Average artificial linear chromosome copy numbers in a population of cells, as measured by quantitative polymerase chain reactions (qPCR), and retention rates, as measured by fluctuation analyses, were performed on a total of 62 individual wild type and *mad2∆* mutant haploid and diploid clones. Wild type cells, both haploids and diploids, displayed phenotypically unique clone-to-clone differences: one group of 15 clones displayed low-copy numbers per cell and high retention rates, were 1 clone was found to have undergone a genomic integration event, and the second group of 15 clones displayed high copy numbers per cell and low retention rates, with the latter values being consistent with the previously published results where only a single clone had been measured. These chromosome states were observed to be unstable when propagated for 10 days under selection, where high copy-low retention rate clones evolved into low copy-high retention rate clones, but no evidence for integration events was observed. By contrast, *mad2∆* haploid and *mad2∆/mad2∆* diploids displayed a suppression of the clone-to-clone differences, where 20 out of 21 clones had mid-level artificial linear chromosome copy numbers per cell, but maintained elevated chromosome retention rates. The elevated levels in retention rates in *mad2∆* and *mad2∆*/*mad2∆* cells were also maintained even in the absence of selection during growth over 3 days. *MAD2/mad2∆* heterozygous diploids displayed multiple clonal groups: 4 with low copy numbers, 5 with mid-level copy numbers, and 1 with a high copy number of artificial linear chromosomes, but all 10 clones uniformly displayed low retention rates. Our observations reveal that *MAD2* function contributes to the ability of yeast cells to maintain a high number of artificial linear chromosomes per cell in some clones, but, counter-intuitively, *mad2∆* suppresses clone-to-clone differences and leads to an improvement in artificial linear chromosome retention rates yielding a more uniform and stable clonal population with mid-level chromosome copy numbers per cell.

## 1. Introduction

There is broad interest in using budding yeast artificial short-linear chromosomes as a tool to study the mechanisms of chromosome segregation in mitosis and as a technology to carry large fragments of exogenous DNA for recombinant protein expression, for bioengineering of entire metabolic pathways, where all of the pathway enzymes are carried on a single large artificial chromosome, or for amplification of exogenous DNA clones, such as fragments of the human or mouse genomes or other organisms [1,2]. Artificial linear chromosomes also have the advantage of containing telomeres, a centromere, and an origin of replication all of which can contribute to a high rate of chromosome segregation fidelity relative to other smaller circular plasmids that lack all of these elements [3,4,5,6,7]. These chromosome elements also allow artificial linear chromosomes to maintain a presence in a population of yeast even in the absence of selection, at least for short durations of time. When working with exogenous DNA elements in microorganisms, such as plasmids, scientists must be mindful of the DNA copy numbers per cell. Both as a tool to study mitosis, and as a technology to carry large fragments of exogenous DNA, the average copy numbers of artificial chromosomes per cell might have an impact on optimal production or yields of desired target biomolecules or biological products such as during large-scale fermentation of yeast cultures. Average linear chromosome numbers in populations of yeast cells have been measured for individual clones in the past using Southern blots with an external piece of plasmid DNA as a reference standard, and retention rates, as measured by classic fluctuation analyses [6,7]. Using these approaches, average copy numbers per yeast cell were calculated as the average chromosome numbers divided by the retention rates, where values in the range of 7.5 artificial linear chromosomes per yeast cell with a retention rate of 88% or 15 artificial linear chromosomes per yeast cell with a retention rate of 61% in haploids were reported [6,7]. One example of a clone with an average of 30 artificial linear chromosomes in a diploid yeast that displayed a retention rate of only 30% has also been reported [7]. These quantitative studies were performed in wild-type yeast cell backgrounds containing all mitotic and cell cycle regulatory pathways which, in principle, should maximize chromosome segregation fidelity, but were limited to single clones [6,7].

To ensure chromosome segregation fidelity, including the segregation of artificial linear chromosomes, eukaryotic cells contain a highly-conserved mitotic spindle checkpoint pathway that promotes accurate chromosome segregation by delaying cell cycle progression until all chromosomes become attached at their kinetochores and are placed under tension between the two sister-kinetochores of paired sister chromatids held together by cohesion rings in opposition to the forces generated by the mitotic spindle before the metaphase-anaphase transition [8,9,10,11]. If a mitotic spindle microtubule attaches to a kinetochore, but physical tension is not built up by the spindle forces between the paired sister kinetochores, the microtubule attachment becomes unstable and is lost, leaving an unattached chromosome, which activates the spindle checkpoint. One core component of the checkpoint pathway is the Mitotic Arrest-Deficient 2 (*MAD2*) gene that ensures chromosome segregation stability so that each daughter cell inherits a single copy of each chromosome [8,9,11]. Mad2 protein is recruited to unattached kinetochores by interacting with the Mitotic Arrest-Deficient 1 (Mad1) protein where it undergoes a dramatic conformational change and becomes bound to Cell Division Cycle 20 (Cdc20) protein [10,11]. This interaction recruits Mitotic Arrest-Deficient 3 (Mad3) and Budding Uninhibited by Benomyl 3 (Bub3) to form the Mitotic Checkpoint Complex (MCC) which blocks the activity of an E3 ubiquitin Ligase, the Anaphase-Promoting Complex/Cyclosome (APC/C) [10,11]. APC/C^Cdc20^ enzyme function is essential to initiate the metaphase-anaphase transition by promoting the ubiquitin-dependent proteolysis of core cell cycle regulators like B-type Cyclins and Securin, an inhibitor of anaphase [10,11]. In budding yeast, the removal of the *MAD2* function destroys spindle checkpoint function. Loss in the Mad2 function creates a failure to form the Mitotic Checkpoint Complex, allowing the APC/C^Cdc20^ to remain active, leading to the pre-mature destruction of B-type Cyclins and Securin which allows progression into anaphase even in the absence of chromosome attachments to the mitotic spindle [10,11].

Short-linear artificial chromosomes present the yeast spindle checkpoint pathway with an additional unique challenge: the forces exerted on the short chromosomes has been proposed to be able to induce premature separation of sister chromatids at a low frequency before the metaphase-anaphase transition, thus making it impossible for the development of tension and proper stable microtubule-chromosome attachments on the pre-maturely separated artificial linear chromosomes, leading into an increase in chromosome mis-segregation rates [12]. Based on our past work, we chose to try and investigate further the consequences of damaging or destroying mitotic spindle checkpoint function on artificial linear chromosome segregation fidelity [12].

Our goal was to investigate the changes in the average number of artificial linear chromosomes per cell arising in response to the loss of *MAD2* spindle checkpoint function in budding yeast. Towards this end, we developed a new quantitative polymerase chain reaction (qPCR) strategy to measure the average artificial linear chromosome copy numbers, and also measured retention rates by classic genetic fluctuation analyses, in haploid and diploid cells where *MAD2* was a wild type, partially reduced or deleted. Initial measurements in wild type *MAD2* cells yielded an unanticipated clone-to-clone difference in chromosome copy numbers from one genetically identical clone compared to another. To explore this clone-to-clone difference in chromosome copy number phenotype further, we expanded our analyses to include 62 individual clones in total and observed that *MAD2* function contributes to the maintenance of a high number of artificial linear chromosomes in some clones, but, counter-intuitively, *mad2∆* haploid and *mad2∆*/*mad2∆* diploid clones display improved artificial chromosome retention rates and suppress clone-to-clone differences in chromosome copy numbers to yield a different phenotype of clones with mid-level chromosome copy numbers per cell with high retention rates.

## 2. Materials and Methods

Standard yeast protocols were performed for the growth and transformation of yeast cells as previously described [13]. The strains used in this study are listed in the Supplementary Materials, and strains and plasmids are available upon request (Table S1). Cells were transformed with circular plasmids as a control, or short-linear artificial chromosomes selected for on complete synthetic media minus leucine (CSM-leu). Circular and linear artificial chromosomes carried either the *LEU2* or *LEU2-∆16* leucine auxotrophic marker for selection on CSM-leu media, where *LEU2-∆16* is a wild type leucine auxotrophic allele containing a 16 base pair deletion upstream of the ATG start codon of the *LEU2* open-reading frame which has no observable effect on mRNA expression levels of the *LEU2* open-reading frame (Figure S1) [7,14,15]. Retention rate assays were performed by growing cells overnight in selective CSM-leu media, and then plating them onto either CSM or CSM-leu plates and counting colonies [6,7]. Briefly, after growth overnight, and OD_600_ was measured to determine the number of cells per milliliter, and then cells were washed and diluted to plate at an approximate density of 100 cells per plate to allow for colony counting. Cells were grown for 2–3 days at 30 °C to allow colonies to grow. Each retention assay measurement for each clone was made 6 times to ensure a low standard deviation and increased confidence in the measured values. Statistical analyses were performed using a GraphPad Prism calculator employing the Student’s unpaired *t*-test (GraphPad, San Diego, CA, USA).

The execution of quantitative polymerase chain reaction (qPCR) chromosome copy number genomic DNA measurements were made by using DNA isolated from cells with TRIzol reagent (ThermoFisher Scientific, Waltham, MA, USA), and qPCR was performed following the manufacturer’s recommends using SYBR GREEN (Roche, San Francisco, CA, USA) and the StepOnePlus Real-Time PCR System (ThermoFisher Scientific, Waltham, MA, USA). Briefly, reaction template DNA and primer concentrations were adjusted, where untransformed wild type haploid (SCSY79) or diploid (SCSY1037) genomic DNA was used as a reference control to obtain Cycle-threshold (Ct) values of about 20 to ensure clear detection of fluorescent SYBR GREEN labeled PCR products as recommended by the manufacturer (ThermoFisher Scientific, Waltham, MA, USA). Multiple combinations of primer pairs were initially tested to determine which pairs gave rise to a single, well-amplified classic PCR product, which is a necessary prerequisite for successful qPCR. Primer pairs selected in this manner for qPCR were “*CUP9* forward” (5′-GCAAAGCCCTGCTACAAATCC-3′) and “*CUP9* reverse” (5′-AGACCTCCTGCCCGAATTTT-3′); “*ILV6*-2 forward” (5′-CAGTGCCGTTTCATCCATCA-3′) and “*ILV6*-2 reverse” (5′-TCTTTGACCTCGGTGTTGCA-3′); “*LEU2*-4 forward” (5′-CATGAGCCACCATTGCCTATT-3’) and “*LEU2*-4 reverse” (5′-TGGCGGCAGAATCAATCAA-3′). Primer pairs were also designed to have identical melting temperatures for qPCR. The “*LEU2-4* forward” and “*LEU2-4* reverse” primer pair amplifies the wild type *LEU2* locus and the endogenous *leu2-3,112* chromosomal mutant locus equally well. To establish the linear range for the qPCR reactions, initial qPCR assays were performed using recombinant DNA plasmids instead of yeast genomic DNA, where we observed that the qPCR measurements remained linear under our conditions within a range of 1–15 measured copies relative to a standard amount of DNA set equal to 1. A typical qPCR reaction contained 1–2 µL of genomic template DNA that had been quantified and tested by classic PCR first to make sure it could serve as a good quality high-PCR yield template, 1.6 µL of the forward primer, 1.6 µL of the reverse primer, 10 µL of the SYBR GREEN reaction mix, where the remaining volume was adjusted to 20 µL with ddH_2_O. If the genomic template DNA was found to be of low quality it was discarded, and new samples were prepared, where each new sample was examined by classic PCR first to ensure its quality as a template yielding only a single well-amplified PCR product. For the initial measurements, qPCR was performed 16 times on genomic DNA samples isolated from each individual haploid clone (8 clones total, meaning 128 individual qPCR reactions) and 12 times on genomic DNA isolated from each individual diploid clone (6 clones total, meaning 72 individual qPCR reactions) to ensure that a calculated final artificial linear chromosome copy number per cell value had a standard deviation of less than 1 (Table S1). However, to measure a larger number of clones (62 artificial linear chromosome clones in total) to further investigate the observed clone-to-clone differences, qPCR reactions were performed only 8 times on the genomic DNA isolated from each individual clone to minimize the use of reagents but still achieve a low standard deviation and high confidence in the measured values for each individual clone sample, where there is in general good agreement between the measurements made using 16, 12, and 8 replicates of the qPCR reactions on the genomic DNA isolated from each clone (Table S2). All data for retention rates, qPCR measurements, and calculated artificial linear chromosome copy numbers are listed for each yeast clone (Table S3).

## 3. Results

### 3.1. Short Linear Artificial Chromosomes Display Clone-to-Clone Differences in Wild Type Haploid Cells

**Figure 1:**
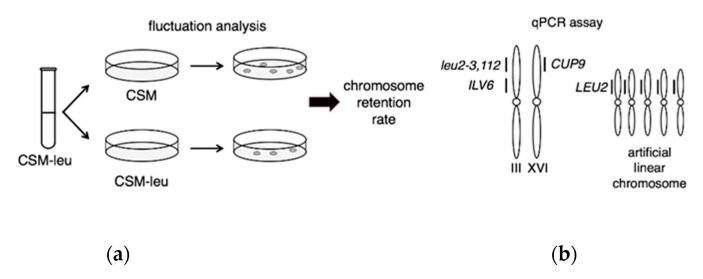

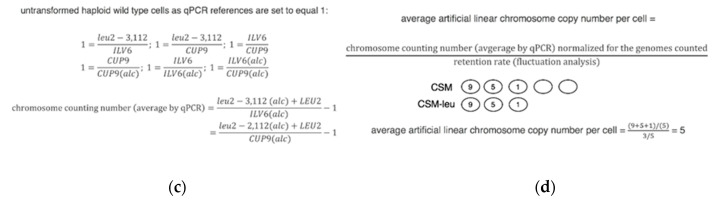
Measurements in a population of yeast of the average artificial chromosome copy numbers per cell: (**a**) Genetic fluctuation assays were performed by initially growing clones in selective CSM-leu media overnight and then plating them on CSM and CSM-leu to measure the average retention rates (3/5 = 60% in this example); (**b**) A schematic diagram of the qPCR assay to measure the number of copies of the *leu2-3,112* + *LEU2* loci present in the isolated genomic DNA from a population of cells. The *IVL6* locus on chromosome III and *CUP9* locus on chromosome XVI served as internal controls that remain at an equal 1:1 ratio with each other and with the endogenous *leu2-3,112* locus, where the small vertical lines represent the qPCR products. PCR primer pairs were designed to ensure amplification of both *leu2-2,112* and *LEU2* at the same level. In this example, the qPCR cycle-threshold (Ct) for the *leu2-3,112* + *LEU2* locus primer pair would occur earlier relative to an untransformed wild type cell population containing only the single *leu2-3,112* locus on chromosome III (the schematic is meant to represent a population of cellular genomic DNA containing an average of 5 artificial linear chromosomes – in a real population made up of many individual cells, some cells would carry more artificial chromosomes and others would carry fewer artificial chromosomes); (**c**) in all qPCR reactions untransformed wild type haploid or diploid cells were employed as reference controls where the ratio between the *leu2-3,112* and *ILV6* and *CUP9* internal controls could be set to equal 1, and then were employed to normalize the measured *ILV6* and *CUP9* signals from qPCR reactions in samples containing artificial linear chromosomes (alc). The average chromosome counting number is defined as the qPCR signal measured relative to the *ILV6* and *CUP9* internal controls, minus 1 in haploids to remove the over-count of the endogenous *leu2-3,112* locus; (**d**) Calculating the average artificial linear chromosome copy numbers per cell. The qPCR amplifies genomic DNA isolated from a population of cells which will include cells that have very recently lost the artificial linear chromosome (for example, in the last mitotic division), even when under selection, but that have not yet died (in this example, 2 cells have lost the artificial chromosomes and 3 cells have retained them; where a total of 20 *leu2-3,112* + *LEU2* loci would be amplified during qPCR off of the genomic DNA of these 5 cells; the over-count of the *leu2-3,112* signal would be subtracted out as described above in (**c**) leaving a total of 15 *LEU2* loci distributed among genomic DNA from 5 cells giving rise to an underestimate of an average of 3 copies of *LEU2* per cell). The retention rate reveals the true number of cells in the population that have retained the artificial chromosome at the moment of isolation of the genomic DNA (3 cells in this example with 9, 5, or 1 copy(ies) of the artificial linear chromosome). The true average copy number of artificial linear chromosomes in the population of cells that have retained the chromosomes is determined by normalizing the measured qPCR average copy number by the number of genomes counted as quantified by the amount of the genomic DNA added as a template to the qPCR reaction sample (5 in this example) divided by the retention rate (3/5 in this example) to yield the final result: an average of 5 copies of the artificial linear chromosomes per cell.

Our initial goal was to investigate the potential role of the mitotic checkpoint gene *MAD2* in the maintenance of artificial linear chromosomes. We employed the classic genetic fluctuation test and developed a qPCR-based method to determine the artificial linear chromosome copy numbers per cell in a population (Figure 1). We initially applied the assay to measure the average number of artificial circular plasmids per cell as a control, where previous reports have established circular chromosomes are carried at 1–2 copies per cell on average [3,4,5,6,7]. Our initial results were in good agreement with this, where average chromosome copy numbers ranged between (0.96 ± 0.23) up to (2.19 ± 0.96) in a variety of wild type *MAD2* and mutant *mad2∆* haploid and diploid clones (Table S1).

**Figure 2:**
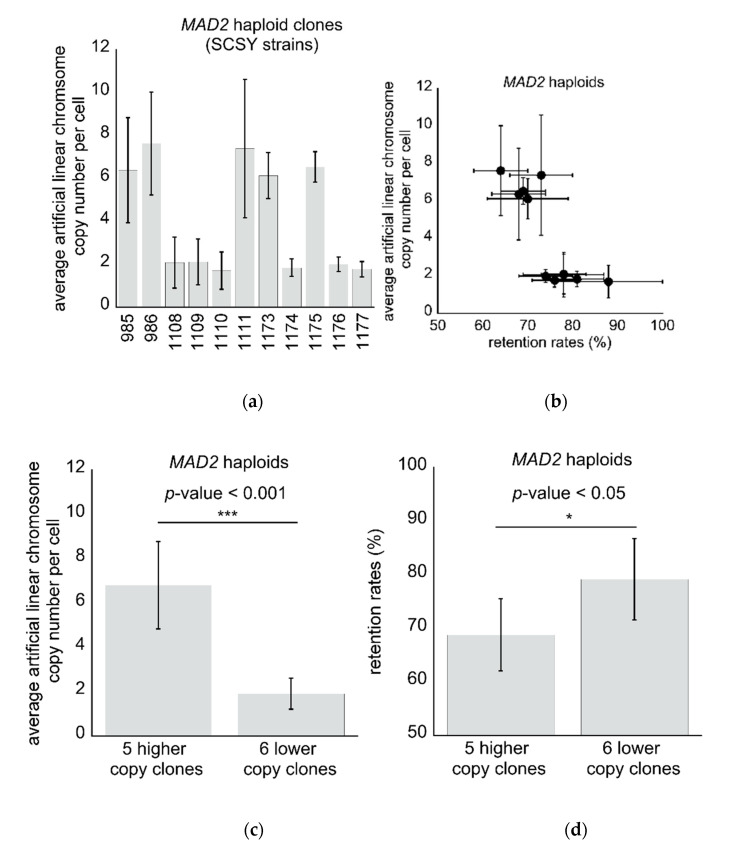
Genetically identical wild type *MAD2* haploid clones have an unexpected phenotype displaying clone-to-clone differences: (**a**) The measured average artificial linear chromosome copy number per cell for 11 clones (*n* = 8 for qPCR; *n* = 6 for retention rates), where the values are displayed as the mean ± standard deviation; (**b**) *MAD2* haploid clones cluster into two phenotypic groups. The graph shows the measured average artificial linear chromosome copy numbers per cell versus the measured retention rates, where values are displayed as the mean ± standard deviation; (**c**) The average artificial linear chromosome copy numbers are significantly different between the higher copy and lower copy clones (*p*-value < 0.001 denoted by ***, Student’s *t*-test); (**d**) The average retention rates are significantly different between the higher copy and lower copy clones (*p*-value < 0.05 denoted by *, Student’s *t*-test).

Having established our assay, we initially measured the average artificial linear chromosome copy numbers in 11 genetically identical wild type haploid clones and detected an unexpected phenotype among the clones (Figure 2). One set of 5 clones displayed a high copy number of artificial linear chromosomes (average of 6.81 ± 1.97) and lower retention rates (average of 68.8 ± 6.6%), while the second set of clones contained a significantly lower copy number of artificial linear chromosomes (average of 1.91 ± 0.71; *p*-value < 0.001) and had significantly higher retention rates (average of 79.2 ± 7.17%; *p*-value < 0.05).

**Figure 3:**
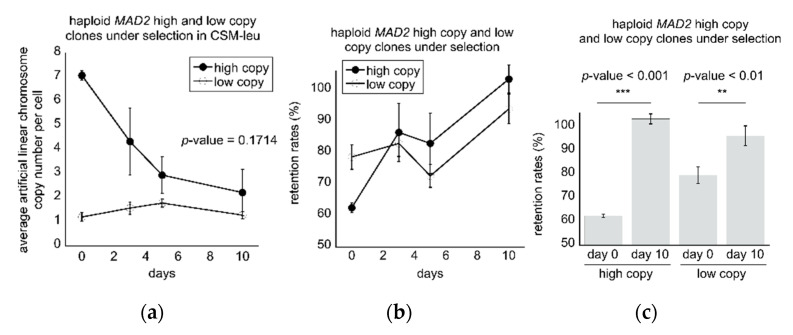
Wild type *MAD2* haploid high copy-low retention rate clones evolved towards low copy-high retention rate clones under CSM-leu selection: (**a**) A comparison of average chromosome copy numbers in 2 high copy-low retention rate *MAD2* clones with 3 low copy-high retention rate *MAD2* clones over 10 days where values are displayed as the mean ± standard deviation. At day 10 there is no significant difference in the artificial chromosome copy numbers per cell (*p*-value = 0.1714, Student’s *t*-test); (**b**) A comparison of 2 high copy-low retention rate *MAD2* clones with 3 low copy-high retention rate *MAD2* clones over 10 days where values are displayed as the mean ± standard deviation; (**c**) Both populations displayed a significant increase in the retention rates by the 10-day point relative to the starting point at day 0 when grown under CSM-leu selection (*p*-values < 0.001 denoted by *** and < 0.01 denoted by ** respectively, Student’s *t*-test).

To characterize clones further, we first measured growth rates by selecting a few clones that have similar retention rates but differed in the average number of artificial linear chromosomes (SCSY1108 and SCSY1111), and clones with somewhat similar artificial linear chromosome copy numbers but different retention rates (SCSY1089 and SCSY1008). However, no change in growth rates were observed relative to wild type control haploid cells (Table S4). We then analyzed 2 wild type high copy clones and 3 low copy clones to determine how stable the chromosome copy numbers and retention rates were over time as the cultures were propagated over 10 days under selection in CSM-leu media. Cultures were diluted and renewed each morning in the presence of the leucine auxotrophic selection (CSM-leu) and the average artificial linear chromosome copy numbers per cell and retention rates were measured at 0, 3, 5, and 10 days. The 2 high copy clones (SCSY985 and SCSY986) displayed a decrease in average copy number per cell over time, while the 3 low copy clones (SCSY1108, SCSY1109, and SCSY1110) maintained the same average low copy number over time, where at 10 days there was no significant difference in chromosome copy numbers between the two groups (*p*-value = 0.1714) (Figure 3a). In addition, all clones displayed an increase in retention rates when grown under selection for 10 days (Figure 3b,c).

One possible explanation for these trends is that the artificial linear chromosomes were integrating into genomic DNA during the course of the 10 days, where the integrated DNA contained the *LEU2* auxotrophic marker. We ruled this out by screening isolated whole-cell DNA for the presence of both arms of the artificial linear chromosome including the telomeres on one side, and also by growing these wild type clones in the absence of selection in CSM and measuring retention rates (Figure S2). If both arms of the artificial linear chromosomes are present including a telomere sequence, as detected by PCR, it would indicate that the artificial chromosome centromere is also present, where the presence of an integrated centromere would create a di-centric chromosome, which is a lethal event. Both arm fragments were detected at day 10 in all 5 clones, and no PCR products were produced by wild type control cells, ruling out genomic integration. In addition, when clones were grown in the absence of leucine auxotrophic selection in CSM, the retention rates in all 5 clones fell to below 10% by 4 days indicating that in wild type cells artificial linear chromosomes were lost when the selective pressure of the auxotrophic marker was removed, which would be the case if the *LEU2* auxotrophic marker had integrated into the genome (Figure S2). These analyses, in combination, suggest that genetically identical wild type *MAD2* haploid cells display clone-to-clone differences in the average copy numbers of artificial linear chromosomes that they carry, but that these differences collapse into a low copy number-high retention rate state when cells are propagated over time under selection.

### 3.2. Mutation in mad2∆ Suppresses Clone-to-Clone Differences in Haploid Cells and Increases Retention Rates Even in the Absence of Selection

**Figure 4:**
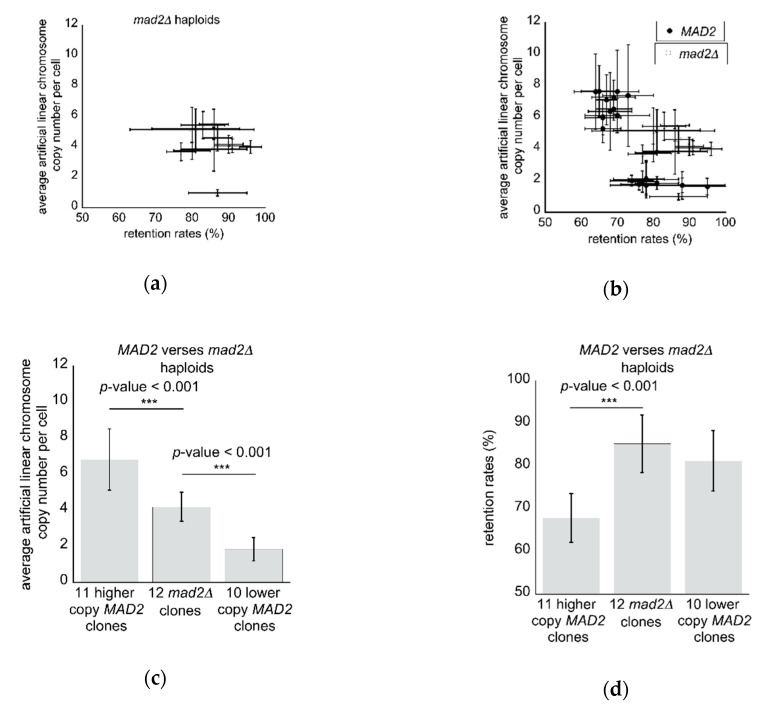
Mutant *mad2∆* haploid clones display suppression of clone-to-clone differences: (**a**) The measured average artificial linear chromosome copy number per cell versus the measured retention rates (*n* = 8 qPCR; *n* = 6 retention rate) where values are displayed as the mean ± standard deviation for 12 *mad2∆* haploid clones; (**b**) A direct comparison between *MAD2* and *mad2∆* haploid clones of average artificial linear chromosome copy number per cell versus the measured retention rates where values are displayed as the mean ± standard deviation; (**c**) The average artificial linear chromosome copy numbers are significantly different between higher copy and lower copy clones of wild type *MAD2* and the *mad2∆* mutant clones (*p*-value < 0.001 denoted by ***, Student’s *t*-test); (**d**) The average retention rates are significantly different between higher copy and *mad2∆* mutant haploids (*p*-value < 0.001 denoted by ***, Student’s *t*-test), but not between the *mad2∆* clones and the lower copy wild type *MAD2* clones.

Having uncovered the unexpected phenotype of clone-to-clone differences in wild type cells, we next investigated a set of 12 mutant *mad2∆* haploid clones to determine the consequences of removing spindle checkpoint function on the artificial linear chromosomes, where an additional 11 *MAD2* haploid clones were also included in these analyses. Mutant *mad2∆* haploid clones suppressed the clone-to-clone differences observed in *MAD2* clones, with 10/11 *mad2∆* clones clustering together in a single mid-level copy number-high retention rate group, with only a single clone displaying a low copy number relative to the rest of the clones (Figure 4a,b). Relative to the high retention rate-low copy *MAD2* clones, *mad2∆* mutant clones had a significantly higher artificial linear chromosome copy number per cell (*p*-value < 0.001) (Figure 4c). Furthermore, *mad2∆* clones displayed a significantly elevated retention rate relative to the high copy-low retention rate *MAD2* clones (*p*-value < 0.001) (Figure 4d).

**Figure 5:**
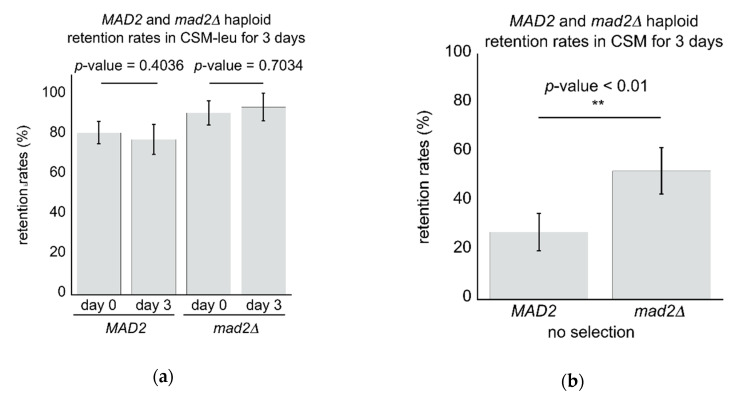
Mutant *mad2∆* cells display a higher retention rate compared to wild type *MAD2* cells even in the absence of auxotrophic selection: (**a**) After 3 days of growth in selective CSM-leu media no significant change in retention rates were observed in either wild type *MAD2* or mutant *mad2∆* cells; (**b**) By contrast, in cells grown for 3 days in CSM in the absence of selection, *mad2∆* cells displayed a significantly higher retention rate for the artificial linear chromosomes (*p*-value < 0.01 denoted by **, Student’s *t*-test).

When culturing cells for large-scale production of biomaterial it may be necessary to change from optimal growth media, which includes factors such as optimal growth rates of the cells, optimal conditions to select for the presence of the artificial linear chromosomes, and the production costs of media raw materials, into a medium designed for optimal induction, production and/or yield of the target biomolecule(s). In such circumstances, it may not be possible to continue selection for the artificial linear chromosome auxotrophy. Thus, we tested directly if *mad2∆* strains continued to display a high retention rate of the artificial linear chromosomes phenotype even in the absence of leucine selection. To this end, *MAD2* and mad2*∆* haploid cells were grown for 3 days in the presence or absence of leucine selection, and chromosome retention rates were measured (Figure 5). In the presence of leucine auxotrophic selection, cells maintained a high retention rate after 3 days, with no significant difference between the starting point retention rates in either wild type *MAD2* or mutant *mad2∆* cells (*p*-value = 0.4036 and 0.7034, respectively). However, in the absence of selection, wild type *MAD2* cell retention rates fell significantly to 27.5%, while mutant *mad2∆* cells only fell to 52.5%. Thus, even in the absence of selection for the artificial linear chromosomes, *mad2∆* mutant cells maintained a significantly higher level of retention compared to wild type *MAD2* cells (*p*-value < 0.01).

### 3.3. Analysis of Diploid Wild Type MAD2/MAD2 and Mutant MAD2/mad2∆ and mad2∆/mad2∆ Clones

**Figure 6:**
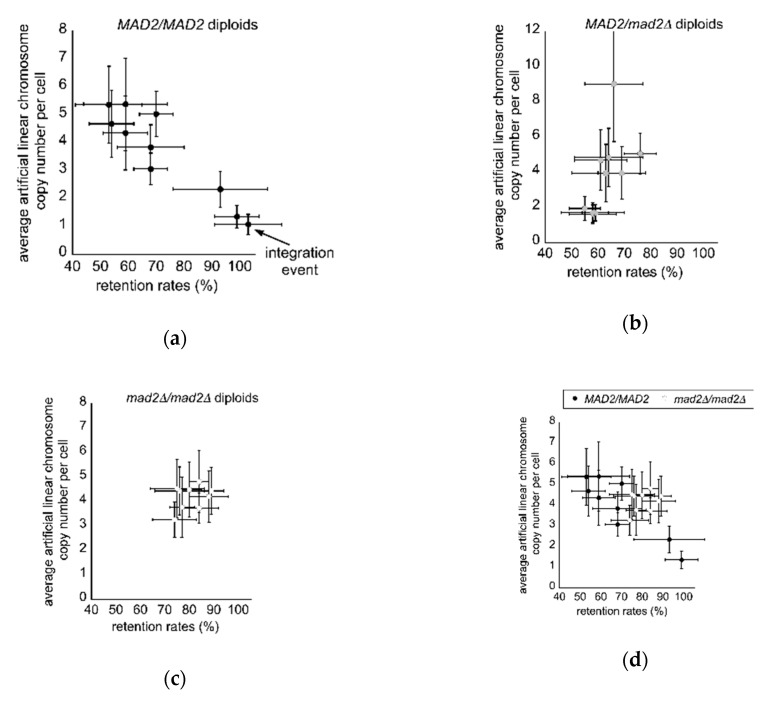

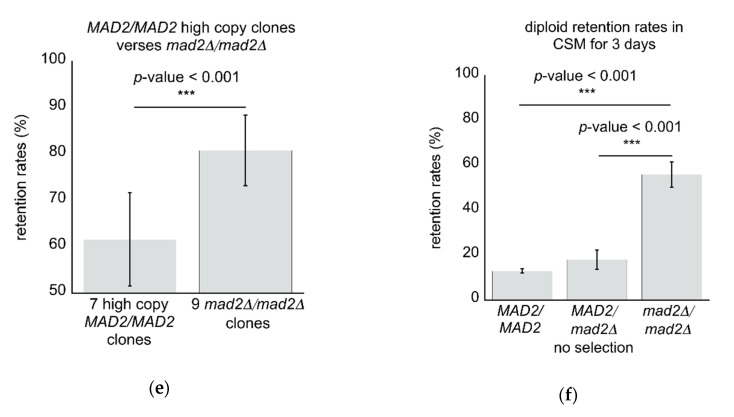
Mutant *mad2∆/mad2∆* diploid clones display suppression of clone-to-clone differences and have high retention rates even in the absence of selection: (**a**) The measured average artificial linear chromosome copy number per cell versus the measured retention rates (*n* = 8 qPCR; *n* = 6 retention rate) where values are displayed as the mean ± standard deviation for 10 *MAD2/MAD2* genetically identical diploid clones. One clone (SCSY1057; marked with an arrow) had undergone an integration event and was excluded from further analyses; (**b**) The measured average artificial linear chromosome copy number per cell versus the measured retention rates (*n* = 8 qPCR; *n* = 6 retention rate) where values are displayed as the mean ± standard deviation for 9 *MAD2/mad2∆* genetically identical diploid clones. All clones uniformly display a low retention rate but separated into high copy, mid-level copy, and low copy groups; (**c**) The measured average artificial linear chromosome copy number per cell versus the measured retention rates (*n* = 8 qPCR; *n* = 6 retention rate) where values are displayed as the mean ± standard deviation for 9 *mad2∆/mad2∆* genetically identical diploid clones that suppress the clone-to-clone differences. All clones cluster into a single group with a mid-level copy number and high retention rates; (**d**) A direct comparison between *MAD2/MAD2* and *mad2∆/mad2∆* haploid clones of average artificial linear chromosome copy number per cell versus the measured retention rates where values are displayed as the mean ± standard deviation; (**e**) the retention rates of artificial linear chromosomes in *mad2∆/mad2∆* are significantly higher compared to wild type *MAD2/MAD2* high copy clones (*p*-values < 0.001 denoted by ***, Student’s *t*-test), even though the chromosome copy numbers are not different (*p*-value = 0.5354, Student’s *t*-test); (**f**) in cells grown for 3 days in CSM in the absence of selection, *mad2∆/mad2∆* cells displayed a significantly higher retention rate for the artificial linear chromosomes compared to both wild type *MAD2/MAD2* and mutant *MAD2/mad2∆* clones in a manner very similar to *mad2∆* haploid clones (*p*-value < 0.001 and < 0.001 denoted by ***, Student’s *t*-test).

In studies of chromosome segregation in mitosis as well as in industrial production settings, diploid budding yeast are often used as a tool for analyses and maximal biomaterial product yield. Thus, we extended our analyses of the artificial linear chromosomes per cell into wild type *MAD2/MAD2* cells as well as mutant *MAD2/mad2∆* and mutant *mad2∆/mad2∆* cells. Wild type *MAD2/MAD2* cells displayed the clone-to-clone difference in a pattern similar to wild type haploids, yielding two phenotypes—one where clones contained high copy numbers and low retention rates, and a second set of clones with low copy numbers and high retention rates (Figure 6a). In these analyses, one clone (SCSY1057) was found to have undergone an integration event that probably occurred shortly after transformation and before the clone was frozen to make a permanent stock, where the chromosome was maintained at a high level even after 3 days of growth in non-selective CSM media - thus, SCSY1057 was excluded from further analysis (marked with an arrow, Figure 6a). One notable difference between wild type haploids and diploids is that the average number of artificial chromosomes in the diploid high copy group (4.54 ± 1.16) is significantly less than that compared to haploid wild type clones (6.82 ± 1.70) (*p*-value < 0.01, Student’s *t*-test). The 10 *MAD2/mad2∆* heterozygous mutant clones displayed a unique distribution: all 10 clones had low retention rates, but the artificial linear chromosome copy numbers fell into 1 of 3 groups, with a low copy number (1.743 ± 0.563), mid-level copy number (4.502 ± 1.544) and a single clone with a high copy number (9.02 ± 3.27) (Figure 6b). Similar to *mad2∆* clones, *mad2∆*/*mad2∆* clones clustered into a single mid-level copy number-high retention rate group, where this group also displayed a significant increase in retention rates compared to the high copy number-low retention rate *MAD2/MAD2* set of clones (*p*-value < 0.001, Student’s *t*-test), even though chromosome copy numbers between the two groups were not different (*p*-value = 0.5354, Student’s *t*-test) (Figure 6c–e). This display of an elevated retention rate in the *mad2∆*/*mad2∆* mutants prompted us to test if these clones would also display an increased retention rate after 3 days of growth in the absence of selection, like *mad2∆* mutant haploid clones. Compared to both wild type *MAD2/MAD2* clones, and *MAD2/mad2∆* mutant clones, the *mad2∆*/*mad2∆* mutant clones displayed a significantly higher retention rate (56.0% ± 5.66%) even in the absence of selection on CSM after 3 days (*p*-value < 0.001 and < 0.001, respectively) (Figure 6f).

## 4. Discussion

These analyses have established that in genetically identical wild type cells there are clone-to-clone differences in artificial short linear chromosome copy numbers per cell and retention rates and that these clonal states are unstable over growth times of 3 days in the absence of selection and 10 days even under selection for the artificial linear chromosomes. Depending on the experimental protocol or biomolecule production process, these clone-to-clone differences could have a significant impact on the outcomes.

In industrial settings, if the goal of a production scientist is to isolate a large amount of an exogenous fragment of DNA hosted in the yeast cell as an insert in a short linear artificial chromosome, the yield could be improved 3 to 4-fold by using haploid cells compared to diploid cells and by measuring the copy numbers in several genetically identical clones first by qPCR to select out the high copy-low retention rate clones [1,2]. However, even if an initial screen is performed to work with a high copy-low retention rate clone, but for biomolecule production cells must be cultured for several days such as during a large volume fermentation run, our results establish that even under selection for the artificial linear chromosome this clonal state will not be stable over an extended period of growth, such as between 3–10 days, and that the clone is likely to evolve towards becoming a low copy-high retention rate population [1,2,16,17]. Thus, in designing a successful high-yield production run the number of days of growth must be minimized. Our results also suggest that if artificial linear chromosomes are to be employed to produce a biomaterial, where in the final stages of production it is necessary to remove media that is optimal for the growth of the cells and the selection of the artificial chromosome and replace it with a media that is optimal for induction or production of the biomolecules, but does not contain the auxotrophic selection, counter-intuitively, it may be better to employ a *mad2∆* or *mad2∆/mad2∆* strain which might have an improved yield based on a higher copy number and higher retention rates even over 3 days in the absence of selection. This suggestion could be tested, for example, by quantitative Western blots against a protein(s) carried only on the artificial linear chromosomes under the control of an inducible promoter that is not under the control of any negative endogenous feedback loop, and comparing directly the protein yield of a high copy clone versus a low copy clone in *MAD2* cells, and/or by comparing the protein yield from a *mad2∆* clone versus a wild type *MAD2* clone after 3-days growth under non-selective media followed by induction of protein expression.

The cellular and molecular mechanism of *mad2∆* suppression of the clone-to-clone differences resulting in higher retention rates remains unknown, but in this regard past research observations may be informative. Artificial short linear chromosomes are proposed to present the mitotic spindle checkpoint with a unique challenge. The lengths of the arms on the chromosomes are short enough that when artificial linear paired sister chromatids experience the mitotic spindle pulling forces at their kinetochores in prophase and metaphase they are sometimes pulled apart prematurely before the metaphase-anaphase transition [12,18]. This pre-mature separation is proposed to have several consequences: (i) the artificial chromosomes cannot maintain attachments to the mitotic spindle, where stable attachments require tension between sister chromatid pairs; (ii) this loss of attachment continuously activates the mitotic spindle checkpoint inducing a cell cycle delay until cells decide to proceed in the cell cycle by “mitotic slippage”; (iii) the unattached artificial chromosomes tend to remain in the mother cells because the mother-bud neck creates a physical barrier against the diffusion of the unattached chromosomes into the daughter cell, which would be reflected as a low retention rate [12,18,19,20]. However, the interpretation of some of these past results may now be confounded by the fact that the number of artificial chromosomes were not measured, so it is unknown for the clones that were employed in these studies if they were high copy-low retention rate clones or low copy-high retention rate clones, where we have observed significant clone-to-clone differences even in genetically identical wild type *MAD2* cells.

**Figure 7:**
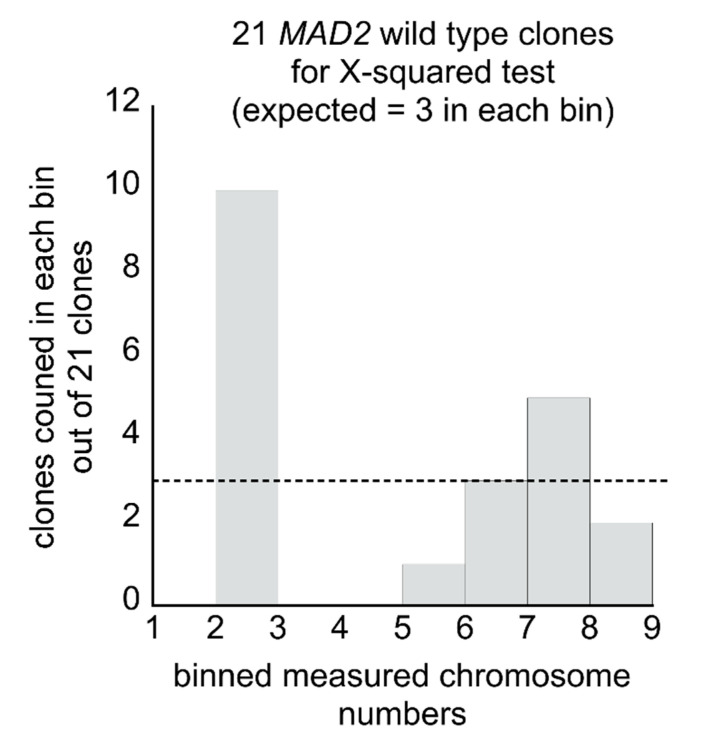
The distribution in average chromosome copy numbers in wild-type *MAD2* clones is not likely to be random: The measured average chromosome copy numbers from 21 wild-type *MAD2* clones are binned into whole number integers by rounding the measured value up or down. The dashed horizontal line represents the average number of 3 clones expected in each group if there was a random distribution of chromosome copy numbers among clones, which in the Χ-squared test is the null hypothesis.

In our current study, for *MAD2* haploid cells, the observed clustering of the clones into two groups gives the appearance of a potential non-random distribution. The number of clones analyzed, 21, is not sufficient to determine the exact nature of the distribution of the clones, but we may have sufficient numbers to reject a simple random distribution. If we assume we are not missing a substantial population of clones that have more chromosomes beyond the upper limit we have observed here, namely, about 8 chromosomes for *MAD2* wild type haploid cells, then we can apply the Χ-squared test (Chi-squared test), where a random equal distribution in chromosome copy numbers among yeast transformants would serve as the null hypothesis. If the measured average chromosome copy numbers are binned together onto integers (by rounding up or down), from our 21 clones, the numbers range from 2–8 chromosomes on average per cell (Figure 7).

The 2–8 chromosomes on average per cell observed means there are 7 expected outcomes if the distribution is random, where for each transformed clone we selected we would expect it to carry between 2–8 chromosomes on average per cell and it would have an equal probability of being anywhere in that range. By Χ-squared, if we made measurements of 21 clones, the null-hypothesis is that we would expect to observe 3 clones of each class equally—with 2, 3, 4, 5, 6, 7, or 8 chromosomes on average per cell. Our experimentally measured Χ-squared value is 28.3. In a system with 7 expected outcomes, there are 6 degrees of freedom, where a Χ-squared value of 28.3 is beyond the critical value necessary as a cut-off to be able to reject the null hypothesis (a cut-off value of 22.458 corresponds to the *p*-value 0.001). However, even if the assumption that cells cannot carry more than 8 chromosomes is true, the rejection of the null hypothesis does not give us any information about what the true distribution might be other than that it is different from random—but, for example, it does not differentiate between a curvilinear distribution versus a bimodal distribution. To make such distinctions measurements on many more clones would need to be made.

We speculate that in wild type *MAD2* or *MAD2/MAD2* cells a transformed clone may start with a low number or a single copy of 1 artificial linear chromosome, but as the single cell grew on the selective media after transformation to make the colony that we picked to place into a permanent stock experienced a pre-mature separation event very early on during colony formation, then after mitotic division, the mother cell would have contained 2 copies and the daughter cell none, where the daughter would not have grown under the auxotrophic selection. This mother cell may have then been a little more likely to experience another pre-mature separation event in subsequent mitosis simply because it was carrying more copies of the artificial chromosome. This may lead to a rapid accumulate 3 chromosomes, and then 4, and then 5 artificial chromosomes by sequential pre-mature separation events—each time becoming more and more likely to suffer another premature separation event in the future as it accumulated more and more artificial chromosomes leading to the bifurcation we observed—some clones with 5–8 chromosomes and others with about 2 chromosomes on average per cell. However, in a competitive population of cells over time, the cells that are accumulating extra chromosomes may ultimately have a selective disadvantage, perhaps when they are carrying about 8 copies of the artificial linear chromosome, which is the upper limit we observed in wild type haploid cells, or 5 copies in wild type diploid cells, where any clone within the population that maintains a low copy of the artificial chromosomes by chance may win out against these unstable and over-burdened cells that have more than about 8 or 5 chromosomes in haploids or diploids respectively.

The highest average copy number per cell we measured was for a single *MAD∆/mad2∆* clone, where these heterozygous mutant cells are most notable for their uniformly low retention rates, suggesting they frequently experience mis-segregation events. *MAD2∆/mad2∆* cells have been demonstrated to have a unique phenotype where they respond normally to loss of attachment events, but they cannot respond specifically to the loss of tension at kinetochores [12]. Thus, there appears to be a correlation between premature loss of tension on short linear chromosomes, and a low retention rate for failing to be able to respond specifically to this loss of tension. We speculate that this instability could lead to a rapid geometric progression in the accumulation of 1, 2, 4, and then 8 artificial linear chromosomes per cell. By contrast, *mad2∆* or *mad2∆/mad2∆* cells simply do not have a spindle checkpoint pathway at all and advance rapidly through the mitotic cell cycle—typically at a rate of 10–15 min faster than wild type cells [12]. This accelerated progression through mitosis can lead to a balance in chromosome segregation and mis-segregation events: the cells do not respond to lack of attachment to the mitotic spindle, leading to the accumulation of the artificial linear chromosomes in the mother cell leading to an elevated average chromosome number per cell, but may also not give the mitotic forces enough time to build upon the paired sister chromatids of artificial linear chromosomes to pull them apart prematurely during the truncated prophase, which would lead to rapid and successful chromosome segregation as reflected by higher retention rates.

Regardless of the underlying mechanisms, our work has empirically established that both basic research scientists and industrial production scientists should be aware of artificial linear chromosome clone-to-clone differences and also aware of how artificial chromosome copy numbers and retention rates can change over time, even under selection. In this study, we only investigated the budding yeast *Saccharomyces cerevisiae*, but the mitotic spindle checkpoint pathway is very highly conserved among all eukaryotes, and the results reported here likely to be applicable to any other budding species of fungi, especially those that contain a constriction at the mother-bud neck during chromosome segregation, including many members of the phylum Ascomycota such as *Pichia pastoris*, Kluyveromyces, Brettanomyces, or *S. bayanus*.

## Supplementary Materials

The following are available online at https://www.mdpi.com/2076-2607/8/10/1495/s1, Figure S1: *LEU2* and *LEU2-∆16* do not have a difference in *LEU2* mRNA expression levels, Figure S2: Classic PCR did not detect any evidence for an integration event by 10 days in any of the 5 clones tested, Table S1: The list of strains used in this study, Table S2: A comparison of performing qPCR 16 or 12 times versus 8 times combined with retention assays performed 6 times to measure artificial chromosome copy numbers per cell, Table S3: All raw data for average artificial chromosome copy numbers per cell measurements, Table S4: Haploid clone growth rates compared to wild type cells.
