# Supplementary material for: Deletion of Budding Yeast MAD2 Suppresses Clone-to-Clone Differences in Artificial Linear Chromosome Copy Numbers and Gives Rise to Higher Retention Rates
Source: Microorganisms. 2020 Sep 29;8(10):1495. doi: 10.3390/microorganisms8101495 (PMC7599710; doi:10.3390/microorganisms8101495)
Supplement: Supplementary file 1 [file microorganisms-08-01495-s001.pdf]

Table S1. The list of strains used in this study [1].

Name (SCSY)	ploidy	*MAD2*	Artificial Chromosome	Artificial Chromosome Copy Number per Cell ± s.d. (n = 16 qPCR haploids; n = 12 diploids)	Artificial Chromosome Copy Number per Cell ± s.d. (n = 8 qPCR)
79	haploid	*MAD2*	-	-	-
569	haploid	*mad2Δ*	-	-	-
1037	diploid	*MAD2/MAD2*	-	-	-
1039	diploid	*MAD2/mad2Δ*	-	-	-
1042	diploid	*mad2Δ/mad2Δ*	-	-	-
987	haploid	*MAD2*	circular *LEU2*	1.86 ± 0.62	-
1005	haploid	*MAD2*	circular *LEU2-Δ16*	2.19 ± 0.69	-
1021	haploid	*mad2Δ*	circular *LEU2*	1.78 ± 0.83	-
1017	haploid	*mad2Δ*	circular *LEU2-Δ16*	2.12 ± 0.81	-
1045	diploid	*MAD2/MAD2*	circular *LEU2*	1.60 ± 0.36	-
1047	diploid	*MAD2/MAD2*	circular *LEU2-Δ16*	1.21 ± 0.29	-
1049	diploid	*MAD2/mad2Δ*	circular *LEU2*	0.96 ± 0.23	-
1051	diploid	*MAD2/mad2Δ*	circular *LEU2-Δ16*	0.97 ± 0.18	-
1067	diploid	*mad2Δ/mad2Δ*	circular *LEU2*	1.33 ± 0.78	-
1053	diploid	*mad2Δ/mad2Δ*	circular *LEU2-Δ16*	1.38 ± 0.61	-
985	haploid	*MAD2*	*LEU2*	6.66 ± 1.43	6.38 ± 2.45
986	haploid	*MAD2*	*LEU2*	6.16 ± 0.44	7.63 ± 2.40
1108	haploid	*MAD2*	*LEU2*		2.08 ± 1.19
1109	haploid	*MAD2*	*LEU2*		2.11 ± 1.06
1110	haploid	*MAD2*	*LEU2*		1.70 ± 0.87
1111	haploid	*MAD2*	*LEU2*		7.39 ± 3.22
1173	haploid	*MAD2*	*LEU2*		6.13 ± 1.07
1174	haploid	*MAD2*	*LEU2*		1.84 ± 0.41
1175	haploid	*MAD2*	*LEU2*		6.54 ± 0.71
1176	haploid	*MAD2*	*LEU2*		2.00 ± 0.34
1177	haploid	*MAD2*	*LEU2*		1.77 ± 0.36
1007	haploid	*MAD2*	*LEU2-Δ16*	2.11 ± 0.92	1.94 ± 0.68
1008	haploid	*MAD2*	*LEU2-Δ16*	1.08 ± 0.22	1.62 ± 0.53
1088	haploid	*MAD2*	*LEU2-Δ16*		1.70 ± 0.47
1189	haploid	*MAD2*	*LEU2-Δ16*		1.71 ± 0.55
1190	haploid	*MAD2*	*LEU2-Δ16*		7.64 ± 2.61
1178	haploid	*MAD2*	*LEU2-Δ16*		7.28 ± 1.07
1179	haploid	*MAD2*	*LEU2-Δ16*		5.99 ± 1.09
1180	haploid	*MAD2*	*LEU2-Δ16*		5.29 ± 0.89
1181	haploid	*MAD2*	*LEU2-Δ16*		7.66 ± 1.62
1182	haploid	*MAD2*	*LEU2-Δ16*		7.11 ± 1.58
1023	haploid	*mad2Δ*	*LEU2*	4.74 ± 1.28	5.15 ± 1.47
1024	haploid	*mad2Δ*	*LEU2*	5.56 ± 1.39	5.19 ± 1.30
1113	haploid	*mad2Δ*	*LEU2*		5.49 ± 0.99
1114	haploid	*mad2Δ*	*LEU2*		5.44 ± 0.89
1115	haploid	*mad2Δ*	*LEU2*		4.18 ± 0.58
1116	haploid	*mad2Δ*	*LEU2*		3.68 ± 0.58
1019	haploid	*mad2Δ*	*LEU2-Δ16*	6.59 ± 1.91	4.60 ± 0.86

1020	haploid	*mad2Δ*	*LEU2-Δ16*	5.52 ± 0.93	3.85 ± 1.44
1118	haploid	*mad2Δ*	*LEU2-Δ16*		4.08 ± 0.45
1119	haploid	*mad2Δ*	*LEU2-Δ16*		0.98 ± 0.21
1120	haploid	*mad2Δ*	*LEU2-Δ16*		4.12 ± 0.43
1121	haploid	*mad2Δ*	*LEU2-Δ16*		3.75 ± 0.55
1055	diploid	*MAD2/MAD2*	*LEU2*	4.58 ± 1.74	5.04 ± 0.81
1056	diploid	*MAD2/MAD2*	*LEU2*		1.36 ± 0.41
1123	diploid	*MAD2/MAD2*	*LEU2*		3.85 ± 0.80
1124	diploid	*MAD2/MAD2*	*LEU2*		2.34 ± 0.64
1125	diploid	*MAD2/MAD2*	*LEU2*		4.36 ± 1.33
1057	diploid	*MAD2/MAD2*	*LEU2-Δ16**	1.25 ± 0.33	1.08 ± 0.36
1058	diploid	*MAD2/MAD2*	*LEU2-Δ16*		3.07 ± 0.57
1103	diploid	*MAD2/MAD2*	*LEU2-Δ16*		5.37 ± 1.38
1104	diploid	*MAD2/MAD2*	*LEU2-Δ16*		5.39 ± 1.66
1105	diploid	*MAD2/MAD2*	*LEU2-Δ16*		4.69 ± 1.20
1059	diploid	*MAD2/mad2Δ*	*LEU2*	3.97 ± 0.82	5.06 ± 1.17
1060	diploid	*MAD2/mad2Δ*	*LEU2*		4.69 ± 1.72
1128	diploid	*MAD2/mad2Δ*	*LEU2*		3.95 ± 1.64
1129	diploid	*MAD2/mad2Δ*	*LEU2*		1.94 ± 0.68
1130	diploid	*MAD2/mad2Δ*	*LEU2*		1.62 ± 0.53
1061	diploid	*MAD2/mad2Δ*	*LEU2-Δ16*	2.04 ± 0.52	1.70 ± 0.47
1062	diploid	*MAD2/mad2Δ*	*LEU2-Δ16*		1.71 ± 0.55
1133	diploid	*MAD2/mad2Δ*	*LEU2-Δ16*		9.02 ± 3.27
1134	diploid	*MAD2/mad2Δ*	*LEU2-Δ16*		4.85 ± 1.65
1135	diploid	*MAD2/mad2Δ*	*LEU2-Δ16*		3.96 ± 1.49
1063	diploid	*mad2Δ/mad2Δ*	*LEU2*	4.65 ± 1.20	3.73 ± 0.62
1064	diploid	*mad2Δ/mad2Δ*	*LEU2*		3.26 ± 0.73
1138	diploid	*mad2Δ/mad2Δ*	*LEU2*		4.21 ± 1.05
1139	diploid	*mad2Δ/mad2Δ*	*LEU2*		3.19 ± 3.45*
1140	diploid	*mad2Δ/mad2Δ*	*LEU2*		3.77 ± 1.24
1065	diploid	*mad2Δ/mad2Δ*	*LEU2-Δ16*	3.67 ± 1.08	4.44 ± 0.98
1066	diploid	*mad2Δ/mad2Δ*	*LEU2-Δ16*		4.82 ± 1.26
1143	diploid	*mad2Δ/mad2Δ*	*LEU2-Δ16*		4.53 ± 1.21
1144	diploid	*mad2Δ/mad2Δ*	*LEU2-Δ16*		4.49 ± 1.13
1145	diploid	*mad2Δ/mad2Δ*	*LEU2-Δ16*		4.44 ± 0.95

[1] All strains are in the w303 strain background: *MATa leu2-3,112 trp1-1 can1-100 ura3-1 ade2-1 his3-11,15* haploids or *MATa/MATα leu2-3,112/leu2-3,112 trp1-1/trp1-1 can1-100/can1-100 ura3-1/ura3-1 ade2-1/ade2-1 his3-11,15/his3-11,15* diploids. The *mad2Δ* alleles are *mad2Δ::URA3, MAD2/mad2Δ::URA3 or mad2Δ::URA3/mad2Δ::kanMX6*. Artificial chromosomes are linear unless noted in the table. *The SCSY1057 strain underwent a chromosomal recombination genomic integration event; the SCSY1139 strain was excluded from analysis because of an excessive error in the qPCR chromosome copy number measurement. s.d. = standard deviation

Table S2. A comparison of performing qPCR 16 or 12 times verses 8 times combined with retention assays performed 6 times to measure artificial chromosome copy numbers per cell [1].

Name (SCSY)	ploidy	Artificial Chromosome Copy Number per Cell ± s.d. (n = 16 qPCR haploids; n = 12 diploids)	Artificial Chromosome Copy Number per Cell ± s.d. (n = 8 qPCR)	*p*-value
985	haploid	6.66 ± 1.43	6.38 ± 2.45	0.8138
986	haploid	6.16 ± 0.44	7.63 ± 2.40	0.1708
1007	haploid	2.11 ± 0.92	1.94 ± 0.68	0.7234
1008	haploid	1.08 ± 0.22	1.62 ± 0.53	0.0439*
1023	haploid	4.74 ± 1.28	5.15 ± 1.47	0.6176
1024	haploid	5.56 ± 1.39	5.19 ± 1.30	0.6442
1019	haploid	6.59 ± 1.91	4.60 ± 0.86	0.0423*
1020	haploid	5.52 ± 0.93	3.85 ± 1.44	0.1180
1055	diploid	4.58 ± 1.74	5.04 ± 0.81	0.5702
1057	diploid	1.25 ± 0.33	1.08 ± 0.36	0.4138
1059	diploid	3.97 ± 0.82	5.06 ± 1.17	0.0912
1061	diploid	2.04 ± 0.52	1.70 ± 0.47	0.2622
1063	diploid	4.65 ± 1.20	3.73 ± 0.62	0.1262
1065	diploid	3.67 ± 1.08	4.44 ± 0.98	0.2250

[1] There is general good agreement between the higher and lower number of qPCR replicates, with only 2/14 samples displaying significant differences (marked with * with *p*-values < 0.05) as determined by Student's *t*-test. s.d. = standard deviation

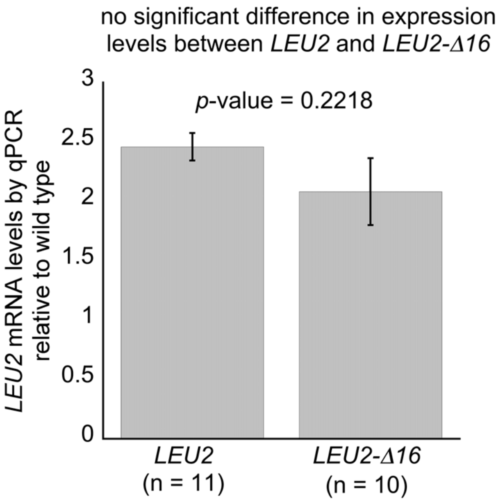

(**a**)

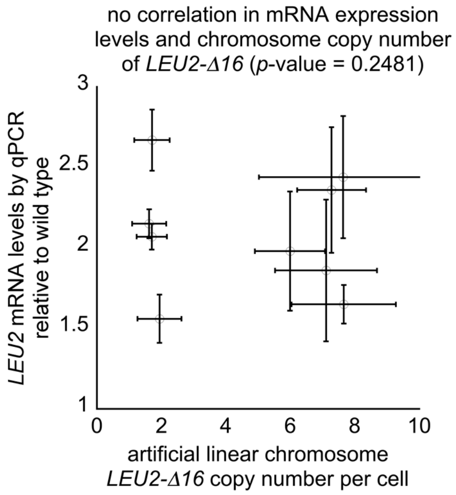

(**b**)

Figure S1. The *LEU2-Δ16* allele is wild type for *LEU2* mRNA expression and does not force cells to carry a high copy number of artificial chromosomes to satisfy leucine auxotrophic selection in CSM-leu media; **(a)** Our original intent was to create artificial linear chromosomes containing the *leu2-d* allele, which had been proposed to have a very low mRNA expression level because of a *Ty*-element insertion upstream of the ATG start site [15] leading to cells being forced to carry a high copy number of chromosomes to complement selective growth in the absence of leucine (CSM-leu) [14,15]. We received a requested plasmid reported to carry the original *leu2-d* allele, but subsequently upon sequencing the plasmid we discovered it only contained an uncharacterized allele of *LEU2* with a 16 base pair deletion upstream of the ATG start site and not the original *Ty*-element insertion mutation (pRS425-Leu2d::ADS) [14]. To characterize this allele, we measured the mRNA expression levels using reverse transcription and qPCR in 11 cells carrying *LEU2* artificial linear chromosomes or in 10 cells carry artificial linear chromosomes marked with the new uncharacterized allele, to measure directly if there was a change in *LEU2* mRNA expression levels. We did not observe any difference in mRNA expression levels between the two (*p*-value = 0.2218, Student's *t*-test). Thus, we named the new allele *LEU2-Δ16* because it displayed wild type levels of *LEU2* mRNA expression; **(b)** Previously, the *leu2-d* allele was shown to force cells to carry a high copy number of circular plasmids or linear artificial chromosomes in order to complement genetic selection on CSM-leu [7,15]. Our analyses of average artificial linear chromosome copy numbers per cell with the *LEU2-Δ16* allele demonstrates there was no correlation between mRNA expression levels and the number of artificial linear chromosomes per cell, and that cells were not forced to exclusively carry a high copy number. Clones containing a low copy number and a high copy number of artificial *LEU2-Δ16* linear chromosomes had the same average *LEU2* mRNA expression levels (*p*-value = 0.2481, Student's *t*-test).

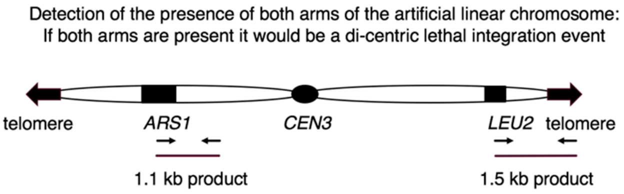

(**a**)

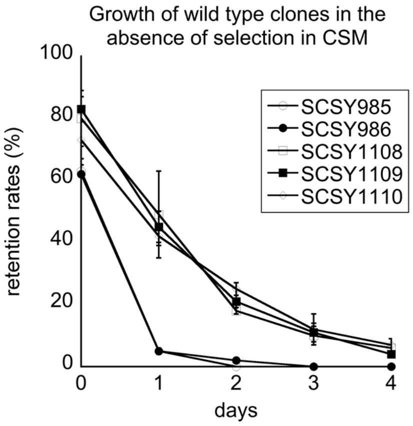

(**b**)

Figure S2. Classic PCR did not detect any evidence for an integration event by 10 days in any of the 5 clones tested. At 10 days, all clones were observed to carry 1-2 copies of the artificial linear chromosome and displayed high retention rates in the range of near 100%. Thus, we tested if these clones had undergone a genomic integration event allowing them to continue to grow under selection; **(a)** The primer design strategy for classic PCR to confirm the artificial linear chromosomes had not integrated. One primer pair amplified a 1.1 kb product on the left arm of the artificial linear chromosome, while a second primer pair amplified a 1.5 kb product off the far-right arm of the chromosome including the *LEU2* marker used for selection and the telomere repeat region. The presence of both PCR products in all 5 clones at the 10-day point indicates the presence of a non-integrated form of the short linear chromosome in the cells. No PCR product was detected in untransformed wild type cells as a control; **(b)** Measurement of retention rates over time in the absence of auxotrophic selection in CSM. All 5 clones displayed a rapid decrease in the artificial linear chromosomes.

Table S3. All data for qPCR average artificial chromosome copy numbers per cell measurements [1].

**Name (SCSY)**	**Artificial Chromosome**	***MAD2***	**Retention Assay (%); average ± s.d.**	**qPCR; average ± s.d.**	**Artificial Chromosome Copy Number per Cell; average ± s.d.**
985	*LEU2*	*MAD2*	59 ± 8 (n=6)	3.90 ± 0.63 (n=16)	6.66 ± 1.43
985	*LEU2*	*MAD2*	68 ± 6 (n=6)	4.34 ± 1.62 (n=8)	6.38 ± 2.45
986	*LEU2*	*MAD2*	59 ± 2 (n=6)	3.62 ± 0.21 (n=16)	6.16 ± 0.44
986	*LEU2*	*MAD2*	64 ± 6 (n=6)	4.92 ± 1.49 (n=8)	7.63 ± 2.40
1007	*LEU2-Δ16*	*MAD2*	65 ± 10 (n=6)	1.37 ± 0.55 (n=16)	2.11 ± 0.92
1008	*LEU2-Δ16*	*MAD2*	95 ± 2 (n=6)	1.02 ± 0.21 (n=16)	1.08 ± 0.22
987	circular *LEU2*	*MAD2*	85 ± 6 (n=6)	1.57 ± 0.51 (n=16)	1.86 ± 0.62
1005	circular *LEU2-Δ16*	*MAD2*	89 ± 6 (n=6)	1.94 ± 0.59 (n=16)	2.19 ± 0.69
1023	*LEU2*	*mad2Δ*	95 ± 3 (n=6)	4.53 ± 1.21 (n=16)	4.74 ± 1.28
1024	*LEU2*	*mad2Δ*	85 ± 7 (n=6)	4.70 ± 1.11 (n=16)	5.56 ± 1.39
1019	*LEU2-Δ16*	*mad2Δ*	79 ± 9 (n=6)	5.20 ± 1.39 (n=16)	6.59 ± 1.91
1020	*LEU2-Δ16*	*mad2Δ*	87 ± 9 (n=6)	4.82 ± 0.63 (n=16)	5.52 ± 0.93
1021	circular *LEU2*	*mad2Δ*	76 ± 19 (n=6)	1.35 ± 0.53 (n=16)	1.78 ± 0.83
1017	circular *LEU2-Δ16*	*mad2Δ*	80 ± 8 (n=6)	1.69 ± 0.63 (n=16)	2.12 ± 0.81
1055	*LEU2*	*MAD2/MAD2*	66 ± 15 (n=6)	3.03 ± 0.90 (n=12)	4.58 ± 1.74
1057	*LEU2-Δ16*	*MAD2/MAD2*	100 ± 5 (n=6)	1.25 ± 0.32 (n=12)	1.25 ± 0.33
1045	circular *LEU2*	*MAD2/MAD2*	89 ± 8 (n=6)	1.43 ± 0.30 (n=12)	1.60 ± 0.36
1047	circular *LEU2-Δ16*	*MAD2/MAD2*	91 ± 4 (n=6)	1.10 ± 0.26 (n=12)	1.21 ± 0.29
1059	*LEU2*	*MAD2/mad2Δ*	77 ± 5 (n=6)	3.05 ± 0.59 (n=12)	3.97 ± 0.82
1061	*LEU2-Δ16*	*MAD2/mad2Δ*	82 ± 3 (n=6)	1.68 ± 0.42 (n=12)	2.04 ± 0.52
1049	circular *LEU2*	*MAD2/mad2Δ*	99 ± 9 (n=6)	0.94 ± 0.21 (n=12)	0.96 ± 0.23

1051	circular *LEU2-Δ16*	*MAD2/mad2Δ*	87 ± 9 (n=6)	0.84 ± 0.13 (n=12)	0.97 ± 0.18
1063	*LEU2*	*mad2Δ/mad2Δ*	82 ± 6 (n=6)	3.80 ± 0.94 (n=12)	4.65 ± 1.20
1065	*LEU2-Δ16*	*mad2Δ/mad2Δ*	93 ± 5 (n=6)	3.43 ± 0.99 (n=12)	3.67 ± 1.08
1067	circular *LEU2*	*mad2Δ/mad2Δ*	80 ± 8 (n=6)	1.06 ± 0.61 (n=12)	1.33 ± 0.78
1053	circular *LEU2-Δ16*	*mad2Δ/mad2Δ*	96 ± 6 (n=6)	1.33 ± 0.58 (n=12)	1.38 ± 0.61
1108	*LEU2*	*MAD2*	78 ± 9 (n=6)	1.62 ± 0.91 (n=8)	2.08 ± 1.19
1109	*LEU2*	*MAD2*	78 ± 5 (n=6)	1.65 ± 0.82 (n=8)	2.11 ± 1.06
1110	*LEU2*	*MAD2*	88 ± 12 (n=6)	1.49 ± 0.74 (n=8)	1.70 ± 0.87
1111	*LEU2*	*MAD2*	73 ± 7 (n=6)	5.38 ± 2.29 (n=8)	7.39 ± 3.22
1173	*LEU2*	*MAD2*	70 ± 9 (n=6)	4.28 ± 0.51 (n=8)	6.13 ± 1.07
1174	*LEU2*	*MAD2*	81 ± 6 (n=6)	1.48 ± 0.31 (n=8)	1.84 ± 0.41
1175	*LEU2*	*MAD2*	69 ± 5 (n=6)	4.49 ± 0.33 (n=8)	6.54 ± 0.71
1176	*LEU2*	*MAD2*	74 ± 6 (n=6)	1.48 ± 0.22 (n=8)	2.00 ± 0.34
1177	*LEU2*	*MAD2*	76 ± 5 (n=6)	1.35 ± 0.26 (n=8)	1.77 ± 0.36
1007	*LEU2-Δ16*	*MAD2*	77 ± 4 (n=6)	1.49 ± 0.52 (n=8)	1.94 ± 0.68
1008	*LEU2-Δ16*	*MAD2*	95 ± 7 (n=6)	1.54 ± 0.49 (n=8)	1.62 ± 0.53
1088	*LEU2-Δ16*	*MAD2*	88 ± 7 (n=6)	1.50 ± 0.40 (n=8)	1.70 ± 0.47
1089	*LEU2-Δ16*	*MAD2*	78 ± 10 (n=6)	1.33 ± 0.39 (n=8)	1.71 ± 0.55
1090	*LEU2-Δ16*	*MAD2*	70 ± 6 (n=6)	5.35 ± 1.78 (n=8)	7.64 ± 2.61
1178	*LEU2-Δ16*	*MAD2*	69 ± 6 (n=6)	5.00 ± 0.59 (n=8)	7.28 ± 1.07
1179	*LEU2-Δ16*	*MAD2*	66 ± 5 (n=6)	3.93 ± 0.65 (n=8)	5.99 ± 1.09
1180	*LEU2-Δ16*	*MAD2*	66 ± 5 (n=6)	3.48 ± 0.53 (n=8)	5.29 ± 0.89
1181	*LEU2-Δ16*	*MAD2*	65 ± 5 (n=6)	4.98 ± 0.97 (n=8)	7.66 ± 1.62
1182	*LEU2-Δ16*	*MAD2*	67 ± 3 (n=6)	4.80 ± 1.04 (n=8)	7.11 ± 1.58
1023	*LEU2*	*mad2Δ*	80 ± 17 (n=6)	4.14 ± 0.80 (n=8)	5.15 ± 1.47
1024	*LEU2*	*mad2Δ*	81 ± 12 (n=6)	4.20 ± 0.85 (n=8)	5.19 ± 1.30

1113	*LEU2*	*mad2Δ*	86 ± 4 (n=6)	4.71 ± 0.82 (n=8)	5.49 ± 0.99
1114	*LEU2*	*mad2Δ*	83 ± 6 (n=6)	4.51 ± 0.65 (n=8)	5.44 ± 0.89
1115	*LEU2*	*mad2Δ*	90 ± 4 (n=6)	3.78 ± 0.50 (n=8)	4.18 ± 0.58
1116	*LEU2*	*mad2Δ*	77 ± 4 (n=6)	2.84 ± 0.43 (n=8)	3.68 ± 0.58
1019	*LEU2-Δ16*	*mad2Δ*	87 ± 4 (n=6)	4.01 ± 0.72 (n=8)	4.60 ± 0.86
1020	*LEU2-Δ16*	*mad2Δ*	86 ± 9 (n=6)	3.32 ± 1.19 (n=8)	3.85 ± 1.44
1118	*LEU2-Δ16*	*mad2Δ*	91 ± 5 (n=6)	3.70 ± 0.34 (n=8)	4.08 ± 0.45
1119	*LEU2-Δ16*	*mad2Δ*	87 ± 8 (n=6)	0.85 ± 0.17 (n=8)	0.98 ± 0.21
1120	*LEU2-Δ16*	*mad2Δ*	96 ± 3 (n=6)	3.95 ± 0.39 (n=8)	4.12 ± 0.43
1121	*LEU2-Δ16*	*mad2Δ*	80 ± 5 (n=6)	2.99 ± 0.40 (n=8)	3.75 ± 0.55
1055	*LEU2*	*MAD2/MAD2*	70 ± 6 (n=6)	3.51 ± 0.48 (n=8)	5.04 ± 0.81
1056	*LEU2*	*MAD2/MAD2*	99 ± 8 (n=6)	1.35 ± 0.39 (n=8)	1.36 ± 0.41
1123	*LEU2*	*MAD2/MAD2*	68 ±12 (n=6)	2.62 ± 0.31 (n=8)	3.85 ± 0.80
1124	*LEU2*	*MAD2/MAD2*	93 ± 17 (n=6)	2.17 ± 0.44 (n=8)	2.34 ± 0.64
1125	*LEU2*	*MAD2/MAD2*	59 ± 8 (n=6)	2.56 ± 0.70 (n=8)	4.36 ± 1.33
1057	*LEU2-Δ16*	*MAD2/MAD2*	103 ± 12 (n=6)	1.11 ± 0.34 (n=8)	1.08 ± 0.36
1058	*LEU2-Δ16*	*MAD2/MAD2*	68 ± 6 (n=6)	2.10 ± 0.34 (n=8)	3.07 ± 0.57
1103	*LEU2-Δ16*	*MAD2/MAD2*	53 ± 12 (n=6)	2.84 ± 0.35 (n=8)	5.37 ± 1.38
1104	*LEU2-Δ16*	*MAD2/MAD2*	59 ± 15 (n=6)	3.16 ± 0.58 (n=8)	5.39 ± 1.66
1105	*LEU2-Δ16*	*MAD2/MAD2*	54 ± 8 (n=6)	2.55 ± 0.54 (n=8)	4.69 ± 1.20
1059	*LEU2*	*MAD2/mad2Δ*	76 ± 6 (n=6)	3.86 ± 0.83 (n=8)	5.06 ± 1.17
1060	*LEU2*	*MAD2/mad2Δ*	61 ± 10 (n=6)	2.86 ± 0.93 (n=8)	4.69 ± 1.72
1128	*LEU2*	*MAD2/mad2Δ*	63 ± 13 (n=6)	2.50 ± 0.90 (n=8)	3.95 ± 1.64
1129	*LEU2*	*MAD2/mad2Δ*	55 ± 6 (n=6)	2.60 ± 0.48 (n=8)	1.94 ± 0.68
1130	*LEU2*	*MAD2/mad2Δ*	58 ± 9 (n=6)	2.04 ± 1.08 (n=8)	1.62 ± 0.53
1061	*LEU2-Δ16*	*MAD2/mad2Δ*	59 ± 12 (n=6)	1.84 ± 0.57 (n=8)	1.70 ± 0.47

1062	*LEU2-Δ16*	*MAD2/mad2Δ*	58 ± 12 (n=6)	2.27 ± 0.78 (n=8)	1.71 ± 0.55
1133	*LEU2-Δ16*	*MAD2/mad2Δ*	66 ± 11 (n=6)	3.53 ± 0.97 (n=8)	9.02 ± 3.27
1134	*LEU2-Δ16*	*MAD2/mad2Δ*	64 ± 13 (n=6)	3.11 ± 0.86 (n=8)	4.85 ± 1.65
1135	*LEU2-Δ16*	*MAD2/mad2Δ*	69 ± 9 (n=6)	2.73 ± 0.96 (n=8)	3.96 ± 1.49
1063	*LEU2*	*mad2Δ/mad2Δ*	84 ± 8 (n=6)	3.15 ± 0.44 (n=8)	3.73 ± 0.62
1064	*LEU2*	*mad2Δ/mad2Δ*	74 ± 9 (n=6)	2.40 ± 0.45 (n=8)	3.26 ± 0.73
1138	*LEU2*	*mad2Δ/mad2Δ*	88 ± 8 (n=6)	3.69 ± 0.86 (n=8)	4.21 ± 1.05
1139	*LEU2*	*mad2Δ/mad2Δ*	78 ± 8 (n=6)	2.49 ± 2.75 (n=8)	3.19 ± 3.45
1140	*LEU2*	*mad2Δ/mad2Δ*	77 ± 5 (n=6)	2.92 ± 0.94 (n=8)	3.77 ± 1.24
1065	*LEU2-Δ16*	*mad2Δ/mad2Δ*	76 ± 10 (n=6)	3.40 ± 0.61 (n=8)	4.44 ± 0.98
1066	*LEU2-Δ16*	*mad2Δ/mad2Δ*	84 ± 4 (n=6)	4.05 ± 1.04 (n=8)	4.82 ± 1.26
1143	*LEU2-Δ16*	*mad2Δ/mad2Δ*	75 ± 11 (n=6)	3.38 ± 0.76 (n=8)	4.53 ± 1.21
1144	*LEU2-Δ16*	*mad2Δ/mad2Δ*	80 ± 5 (n=6)	3.57 ± 0.87 (n=8)	4.49 ± 1.13
1145	*LEU2-Δ16*	*mad2Δ/mad2Δ*	89 ± 5 (n=6)	3.96 ± 0.82 (n=8)	4.44 ± 0.95

[1] Artificial chromosomes are linear unless noted in the table. .s.d. = standard deviation

Table S4. Haploid clone growth rates compared to wild type cells [1].

Name (SCSY)	Retention Rate ± s.d.	Average Artificial Chromosome Copy Number per Cell ± s.d.	Growth Rates (hours) ± s.d. (n = 4)	*p*-value Compared to Untransformed Wild Type
1108	78% ± 9%	2.08 ± 1.19	1.867 ± 0.0973	0.0868
1111	73% ± 7%	7.39 ± 3.22	2.052 ± 0.0732	0.1625
1008	95% ± 7%	1.62 ± 0.53	1.978 ± 0.2089	0.7801
1089	78% ± 10%	1.71 ± 0.55	2.0188 ± 0.160	0.9645

[1] *p*-values as determined by Student's *t*-test. s.d. = standard deviation
